# CAR Treg synergy with anti-CD154 promotes infectious tolerance and dictates allogeneic heart transplant acceptance

**DOI:** 10.1172/jci.insight.188624

**Published:** 2025-04-08

**Authors:** Samarth S. Durgam, Isaac Rosado-Sánchez, Dengping Yin, Madeleine Speck, Majid Mojibian, Ismail Sayin, Grace E. Hynes, Maria-Luisa Alegre, Megan K. Levings, Anita S. Chong

**Affiliations:** 1 Department of Surgery, University of Chicago, Chicago, Illinois, USA.; 2 BC Children’s Hospital Research Institute, Vancouver, British Columbia, Canada.; 3 Department of Surgery, University of British Columbia, Vancouver, British Columbia, Canada.; 4 Department of Medicine, University of Chicago, Chicago, Illinois, USA.; 5 School of Biomedical Engineering, University of British Columbia, Vancouver, British Columbia, Canada.

**Keywords:** Immunology, Therapeutics, Transplantation, Tolerance

## Abstract

Successful allograft-specific tolerance induction would eliminate the need for daily immunosuppression and improve posttransplant quality of life. Adoptive cell therapy with regulatory T cells expressing donor-specific chimeric antigen receptors (CAR Tregs) is a promising strategy but, as monotherapy, cannot prolong survival with allografts with multiple MHC mismatches. Using an HLA-A2–transgenic haplo-mismatched heart transplantation model in immunocompetent C57BL/6 recipients, we showed that HLA-A2–specific CAR (A2.CAR) Tregs were able to synergize with a low dose of anti-CD154 to enhance graft survival. Using haplo-mismatched grafts expressing the 2W-OVA transgene and tetramer-based tracking of 2W- and OVA-specific T cells, we showed that in mice with accepted grafts, A2.CAR Tregs inhibited donor-specific T cell, B cell, and antibody responses and promoted a substantial increase in endogenous FOXP3^+^ Tregs with indirect donor specificity. By contrast, in mice where A2.CAR Tregs failed to prolong graft survival, FOXP3^–^ A2.CAR T cells preferentially accumulated in rejecting allografts, and endogenous donor-specific responses were not controlled. This study therefore provides evidence for synergy between A2.CAR Tregs and CD154 blockade to promote infectious tolerance in immunocompetent recipients of haplo-mismatched heart grafts and defines features of A2.CAR Tregs when they fail to reshape host immunity toward allograft tolerance.

## Abstract 2

This study uses a multiple antigen mismatch mouse heart transplant model to investigate the mechanisms by which CAR-Tregs synergize with CD40 costimulation blockade to prolong graft survival. We demonstrate that, under conditions of prolonged graft survival, A2-specific CAR-Tregs stably express FoxP3 and effectively control Teff cells of non-A2 specificities. Conversely, when graft survival was not prolonged, FoxP3-negative CAR-Tregs accumulate within the graft and failed to protect the allograft.

## Graphical abstract

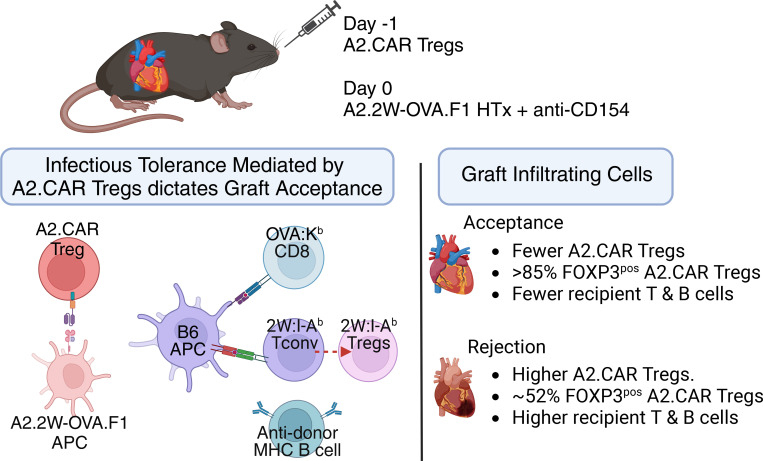

## Introduction

Inducing allograft-specific tolerance would reduce the need for daily pharmacological immunosuppression and its associated side effects, as well as improve long-term allograft survival. One way to induce tolerance is combined transplantation of hematopoietic stem cells with solid organ allografts; while this has resulted in some clinical success, conditioning regimen intensity and risk of graft-versus-host disease limit its broad application (1–3). Another strategy is to harness the natural immunosuppressive properties of FOXP3-expressing regulatory T cells (Tregs) through adoptive cell therapy (2, 3). This approach can support the survival of multiple-antigen-mismatched allografts, via the ability of Tregs to modulate antigen-presenting cells (APCs) and promote the development of Tregs with multiple donor specificities through a process known as infectious tolerance (4, 5). Building upon evidence in mice that infusion of polyclonal, expanded Tregs can induce transplantation tolerance (6–8), several phase I trials have tested the effects of infused polyclonal Tregs in solid organ or stem cell transplantation. These studies have uniformly shown that the approach is feasible, is safe, and may allow a reduction of pharmacological immunosuppression (8–11).

One limitation of cell therapy with polyclonal Tregs is that only a small proportion are alloantigen specific. Work in preclinical models of transplantation has shown that antigen-specific Tregs are significantly more effective than are polyclonal cells (12). Alloantigen-specific Tregs can be generated by repetitive stimulation with donor-derived APCs (13–16), or donor antigen specificity can be introduced into Tregs by expression of chimeric antigen receptors (CARs) (9, 17). Several groups have expressed HLA-A2–specific CARs (A2.CARs) in human Tregs and shown that they have superior therapeutic effects over polyclonal Tregs in humanized mouse models of graft-versus-host disease or skin transplantation (18, 19). These observations have led to a rapid clinical translation of this approach (ClinicalTrials.gov NCT05234190, NCT04817774).

To dissect mechanistic effects and iteratively improve efficacy, A2.CAR mouse Tregs have recently been studied in mouse transplant models and reported to delay rejection of A2-mismatched skin grafts and inhibit A2-specific antibody formation (20, 21). Similarly, in a model of A2-mismatched heart transplantation (HTx), A2.CAR Tregs significantly promoted graft survival over polyclonal Tregs, though graft palpation scores started to decline by day 20 post-HTx. In a haplo-mismatched A2.C57BL/6 (B6) × BALB/c (A2.F1) model of HTx, A2.CAR Treg monotherapy was ineffective, and significant extension of allograft survival was observed only when combined with 9 days of rapamycin treatment (22). Notably, this combination did not induce long-term graft acceptance, highlighting the need for further work to identify therapies capable of synergizing with CAR Tregs to achieve stable transplantation tolerance.

We previously reported that costimulation blockade with cytotoxic T lymphocyte antigen 4 Ig (CTLA4-Ig) and rapamycin prevented the expansion of both endogenous donor-specific FOXP3^neg^CD4^+^ T conventional cells (Tconvs) and Tregs. On the other hand, anti-CD154 permitted Treg expansion while inhibiting that of Tconvs (23), consistent with the fact that Tregs express lower levels of CD154 compared with Tconvs (24). Given the resurgence of interest in clinical use of modified versions of anti-CD154 without pro-thrombotic properties (25, 26) (NCT04046549, NCT05983770), in this study, we tested whether anti-CD154 could enhance the pro-tolerogenic properties of CAR Tregs in a clinically relevant haplo-mismatched (A2.2W-OVA.F1) HTx model in immunocompetent B6 recipients. We showed that A2.CAR Tregs synergized with anti-CD154 to enhance graft survival and induce infectious tolerance. Specifically, expansions of endogenous donor-reactive CD4^+^FOXP3^neg^ Tconvs and CD8^+^ T cells and endogenous donor-specific antibody (DSA) responses to non-A2 alloantigens were inhibited, and the expansion of endogenous donor-reactive CD4^+^FOXP3^pos^ Tregs was promoted. In contrast, when A2.CAR Tregs failed to promote graft acceptance, infectious tolerance was not observed.

## Results

### A2.CAR Tregs synergize with low-dose anti-CD154 to prolong survival of haplo-mismatched heart grafts.

**Figure 1:**
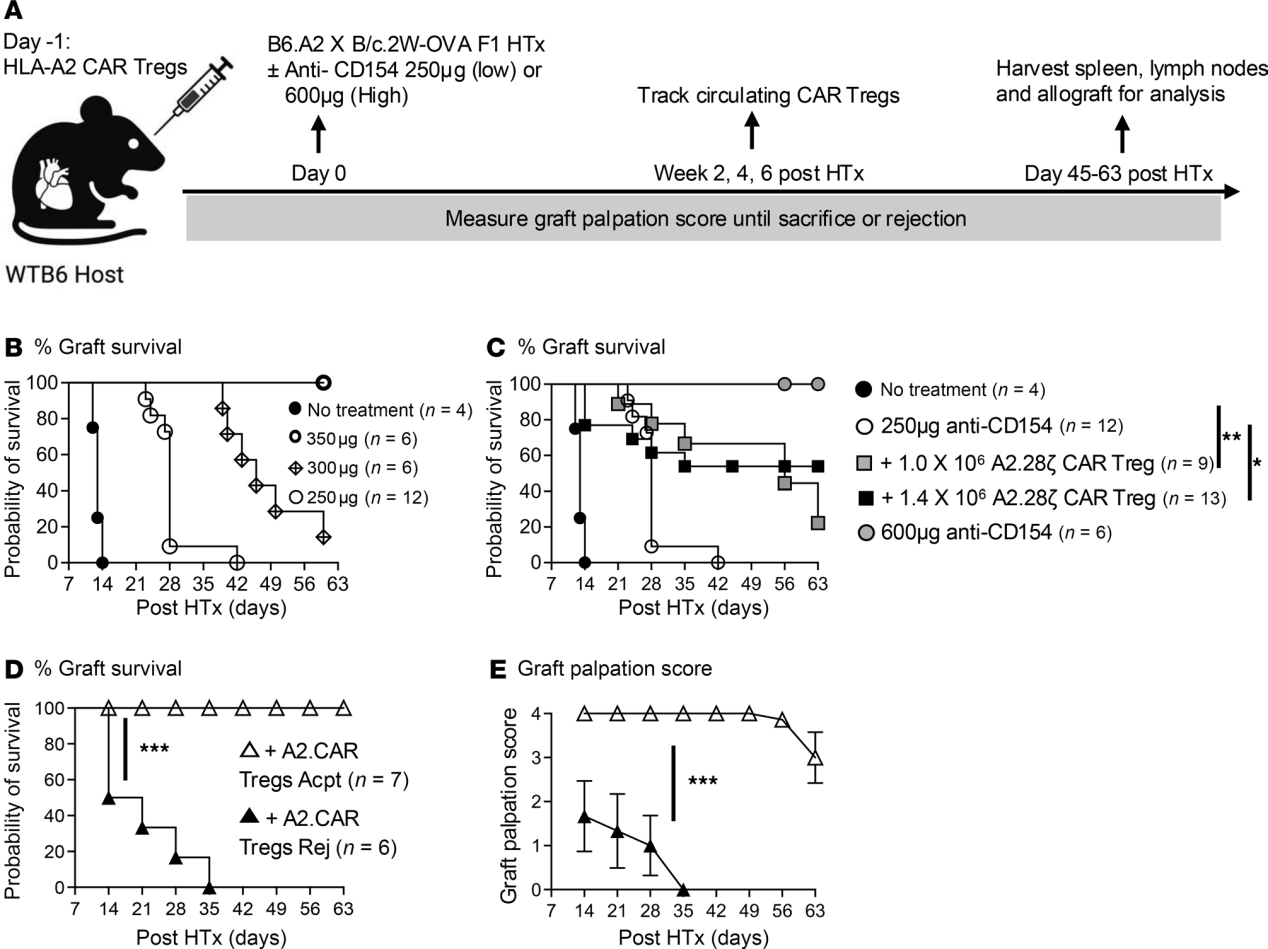
A2.CAR Tregs synergize with low-dose anti-CD154 to prolong the survival of haplo-mismatched heart grafts. (**A**) Schematic of the experimental model. (**B**) Graft survival curve in recipients of A2.2W-OVA.F1 heart transplantation (HTx) with 3 different doses of anti-CD154. (**C**) Graft survival of B6 recipients of A2.2W-OVA.F1 HTx treated with low anti-CD154 (250 μg) without or with CAR Tregs or high anti-CD154 (600 μg). Survival curve for recipients receiving only 250 μg anti-CD154 is the same in **B** and **C**. Graft survival (**D**) and palpation scores (**E**) in recipients of 1.4 × 10^6^ A2.CAR Tregs + low anti-CD154 with rejected (Rej) or accepted (Acpt) grafts. Symbols are the same for **D** and **E**. Log-rank (Mantel-Cox) test for statistical significance. Each symbol represents 1 mouse. Data are presented as mean ± SEM, and statistical significance was assessed by Mann-Whitney test. *P < 0.05; **P < 0.01; ***P < 0.001.

A second-generation, HLA-A2–specific CAR encoding a CD28 costimulatory domain (Supplemental Figure 1A; supplemental material available online with this article; https://doi.org/10.1172/jci.insight.188624DS1) was retrovirally transduced into polyclonally stimulated CD4^+^CD8^–^Thy1.1^+^FOXP3^gfp^ Tregs. CAR transduction was confirmed on the basis of expression of the CAR-encoded extracellular c-Myc tag. The resulting A2.CAR Tregs were at least 95% FOXP3^+^ (Supplemental Figure 1, B and C). Previous reports by Wagner et al. showed that A2.CAR Treg monotherapy failed to promote the survival of haplo-mismatched HTx and that adjunct immunosuppression was necessary (22). Our previous observation that anti-CD154 was superior to CTLA4-Ig or rapamycin in permitting expansion of endogenous donor-reactive Tregs (23) prompted us to test if a suboptimal dose (250 μg/mouse) of anti-CD154 would synergize with A2.CAR Tregs in B6 recipients of haplo-mismatched HTx (Figure 1A). While untreated recipients acutely rejected their heart allografts with a median graft survival of 12 days, an anti-CD154 dose of 250 μg/mouse was chosen, as it delayed rejection to a median survival of 28 days, whereas a higher dose of 350 μg anti-CD154 uniformly induced graft acceptance (Figure 1B).

Recipients of 1.0 × 10^6^ A2.CAR Tregs + anti-CD154 had significantly prolonged median survival of 56 days, as did recipients of a higher dose of 1.4 × 10^6^ A2.CAR Tregs (henceforth referred to as +A2.CAR Tregs) (Figure 1C). However, there was considerable heterogeneity in graft survival (Figure 1, D and E), with 23% (*n* = 3/13) rapidly rejecting their grafts by day 14 and 23% (*n* = 3/13) by day 35 post-HTx. The remaining 53% of recipients (*n* = 7/13) maintained a graft palpation score of more than 3 until the day of sacrifice (day 45–63 post-HTx). Thus, +A2.CAR Treg recipients were grouped into Rej and Acpt groups for subsequent characterization of A2.CAR Tregs and endogenous donor-specific T and B cell responses.

### Rejection in A2.CAR Treg recipients is associated with reduced FOXP3^pos^/FOXP3^neg^ A2.CAR T cell ratios within heart allografts.

**Figure 2:**
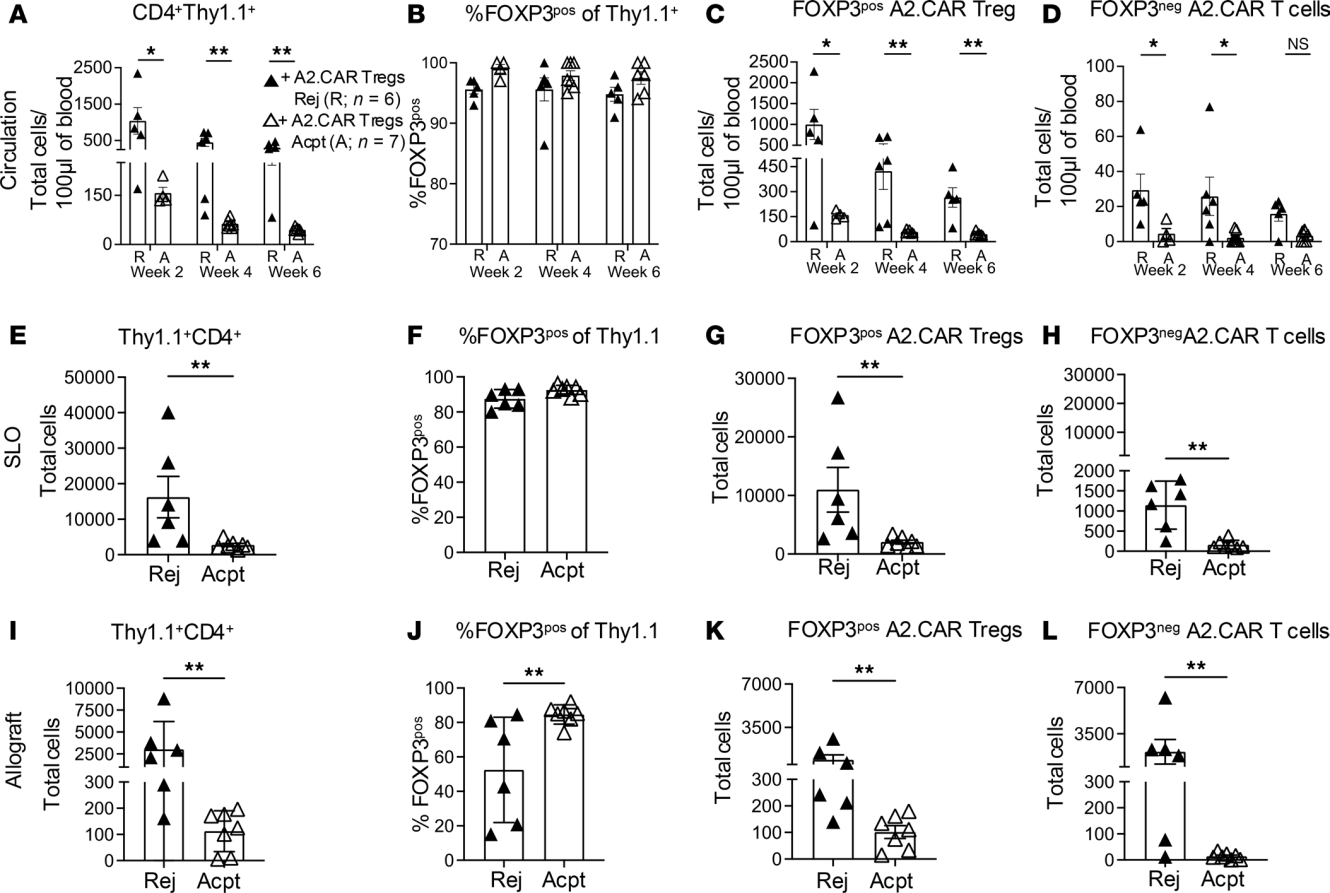
Recipients of A2.CAR Tregs and low-dose anti-CD154 with Acpt allografts have low accumulation of CD4^+^Thy1.1^+^ cells and high ratios of FOXP3^pos^/FOXP3^neg^ A2.CAR T cells. (**A**–**D**) The absolute numbers and proportions of circulating Thy1.1^+^ A2.CAR Tregs in A2.CAR Treg recipients with Rej (filled triangles) or Acpt (unfilled triangles) grafts was quantified at the indicated weeks (**A**–**D**) or at the experimental endpoint of postoperative day (POD) 45–63 (**E**–**L**). (**A**) Number of CD4^+^Thy1.1^+^ cells per 100 μL blood. (**B**) Percentage FOXP3^pos^ of Thy1.1^+^ cells. (**C** and **D**) Number of FOXP3^pos^ or FOXP3^neg^ A2.CAR T cells per 100 μL blood. (**E**–**L**) At the experimental endpoint (POD 45–63), SLOs and allografts were harvested, and Thy1.1^+^ A2.CAR Tregs were quantified. (**E** and **I**) Total number of CD4**^+^**Thy1.1**^+^** T cells from SLOs and allografts from Rej (*n* = 6) or Acpt (*n* = 7) recipients. (**F** and **J**) Percentage FOXP3^pos^ of Thy1.1^+^ cells. Number of FOXP3^pos^ (**G** and **K**) or FOXP3^neg^ (**H** and **L**) A2.CAR T cells recovered from SLOs and allografts. Each symbol represents 1 mouse. Data are presented as mean ± SEM, and statistical significance was assessed by Mann-Whitney test. *P < 0.05; **P < 0.01. Total number of CD4^+^Thy1.1^+^ cells from SLOs normalized to 3 × 10^6^ events. Total number of CD4^+^Thy1.1^+^ cells from allografts normalized to 1 × 10^6^ events.

Heterogeneity in graft survival in A2.CAR Treg recipients prompted us to track circulating CD4^+^Thy1.1^+^ A2.CAR Tregs at weeks 2–6 post-HTx (Supplemental Figure 1D). At all time points, a ~10-fold higher number of CD4^+^Thy1.1^+^ A2.CAR Tregs was detected in the blood of Rej compared with Acpt recipients (Figure 2A), though the percentage expressing FOXP3 in Rej versus Acpt recipients was not significantly different across all 3 time points (Figure 2B). Consequently, both FOXP3^pos^ and FOXP3^neg^ circulating A2.CAR T cells were present in higher numbers in Rej compared with Acpt recipients (Figure 2, C and D). This finding suggests that an early expansion of circulating A2.CAR Tregs was unexpectedly associated with poor allograft survival.

To verify the association between expanded numbers of A2.CAR Tregs and poor allograft survival, we quantified FOXP3^pos^ and FOXP3^neg^ of CD4^+^Thy.1^+^ cells from the spleen and lymph nodes (secondary lymphoid organs, SLOs) as well as allografts at the time of sacrifice (day 45–63 post-HTx) (Figure 2, E–L). Similar to blood, significantly more (6- to 7-fold) CD4^+^Thy1.1^+^ CAR Tregs were recovered from the SLOs of Rej versus Acpt recipients, with comparable percentages of FOXP3^pos^ of CD4^+^Thy1.1^+^ cells (87%–93%). Thus, more FOXP3^pos^ and FOXP3^neg^ A2.CAR T cells were recovered from Rej versus Acpt recipients (Figure 2, E–H, and Supplemental Figure 2A). CAR (Myc) expression was also significantly elevated on A2.CAR Tregs from the circulation and SLOs of Rej versus Acpt recipients (Supplemental Figure 2, B–E).

The number of total CD4^+^Thy1.1^+^ cells infiltrating Rej allografts was also higher (27- ± 28-fold) compared with Acpt recipients (Figure 2I). Notably, only 52% ± 30% of Thy1.1^+^ cells were FOXP3^pos^ in Rej compared with 85% ± 5% in Acpt allografts (Figure 2J). As a result, the recovered FOXP3^pos^ cells were only a mean of 8-fold (1,880 ± 2,492) higher whereas FOXP3^neg^ cells were ~130-fold higher (3,562 ± 4,368) in Rej allografts compared with Acpt (Figure 2, K and L). Finally, while the overall CAR (Myc) expression by graft-infiltrating CD4^+^Thy1.1^+^ T cells was lower compared with those in blood or SLO, CAR expression was significantly higher from Rej versus Acpt allografts (Supplemental Figure 2, F and G).

### FOXP3^pos^ and FOXP3^neg^ A2.CAR T cells acquire distinct activation phenotypes in rejection and acceptance in the SLOs.

**Figure 3:**
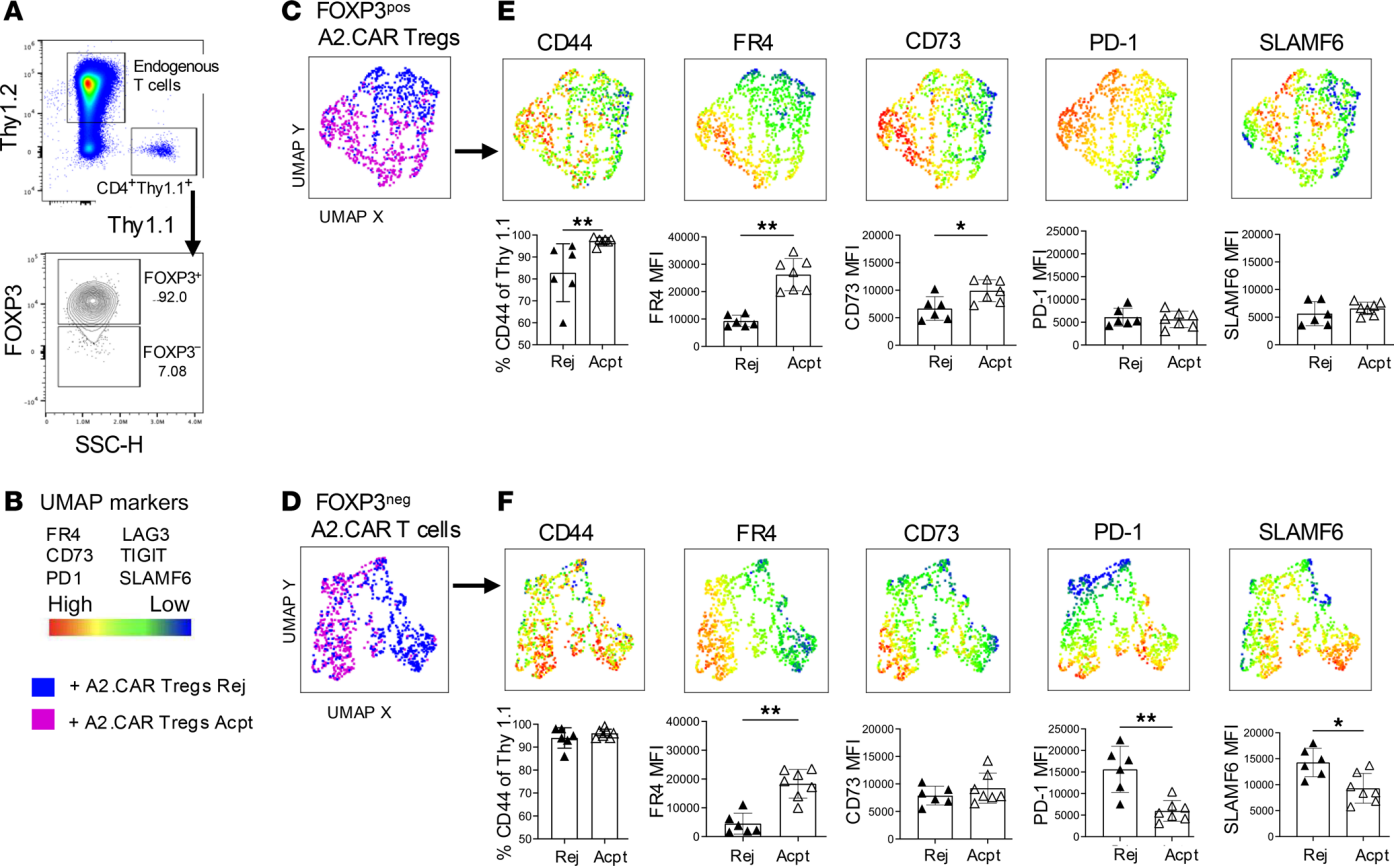
FOXP3^pos^ and FOXP3^neg^ A2.CAR T cells in the SLOs are phenotypically distinct in recipients with Rej or Acpt allografts. (**A**) Gating strategy to define FOXP3^pos^ and FOXP3^neg^ cells within CD4^+^Thy1.1^+^ A2.CAR T cells from SLOs (POD 45–63). (**B**) UMAP markers and experimental groups. (**C** and **D**) UMAP plots demonstrating phenotypic differences in (**E**) FOXP3^pos^ and (**F**) FOXP3^neg^ A2.CAR T cells from Rej and Acpt recipients, based on expression of FR4, CD73, PD1, SLAMF6, LAG3, and TIGIT. UMAP with heatmap and bar plots showing relative expression of indicated markers based on normalized median fluorescence intensity (MFI). Each symbol in the bar plots represents 1 mouse. Data are presented as mean ± SEM, and statistical significance was determined by Mann-Whitney test. *P < 0.05; **P < 0.01.

The differential expansion of FOXP3^pos^ versus FOXP3^neg^ A2.CAR T cells in Rej versus Acpt recipients prompted a phenotypic analysis of markers associated with Treg function or T cell dysfunction, namely FR4, CD73, PD1, SLAMF6, LAG3, and TIGIT. Uniform manifold approximation and projection (UMAP) was used to visualize the phenotype at single-cell resolution (Figure 3, A and B), revealing that FOXP3^pos^ and FOXP3^neg^ A2.CAR T cells from Acpt versus Rej recipients were phenotypically distinct (Figure 3, C–F). Specifically, FOXP3^pos^ A2.CAR Tregs from Acpt recipients had higher expression of CD44, FR4, and CD73 but comparable levels of PD-1 and SLAMF6 (Figure 3E). In contrast, A2.CAR T cells in Rej recipients exhibited significantly greater expansion and acquired a phenotype suggestive of reduced regulatory function. FOXP3^neg^ A2.CAR T cells from Acpt versus Rej recipients also expressed higher FR4 but lower PD-1 and SLAMF6 (Figure 3F).

### A2.CAR Tregs promote endogenous Treg expansion and suppress donor-reactive Tconvs and CD8^+^ T cells.

**Figure 4:**
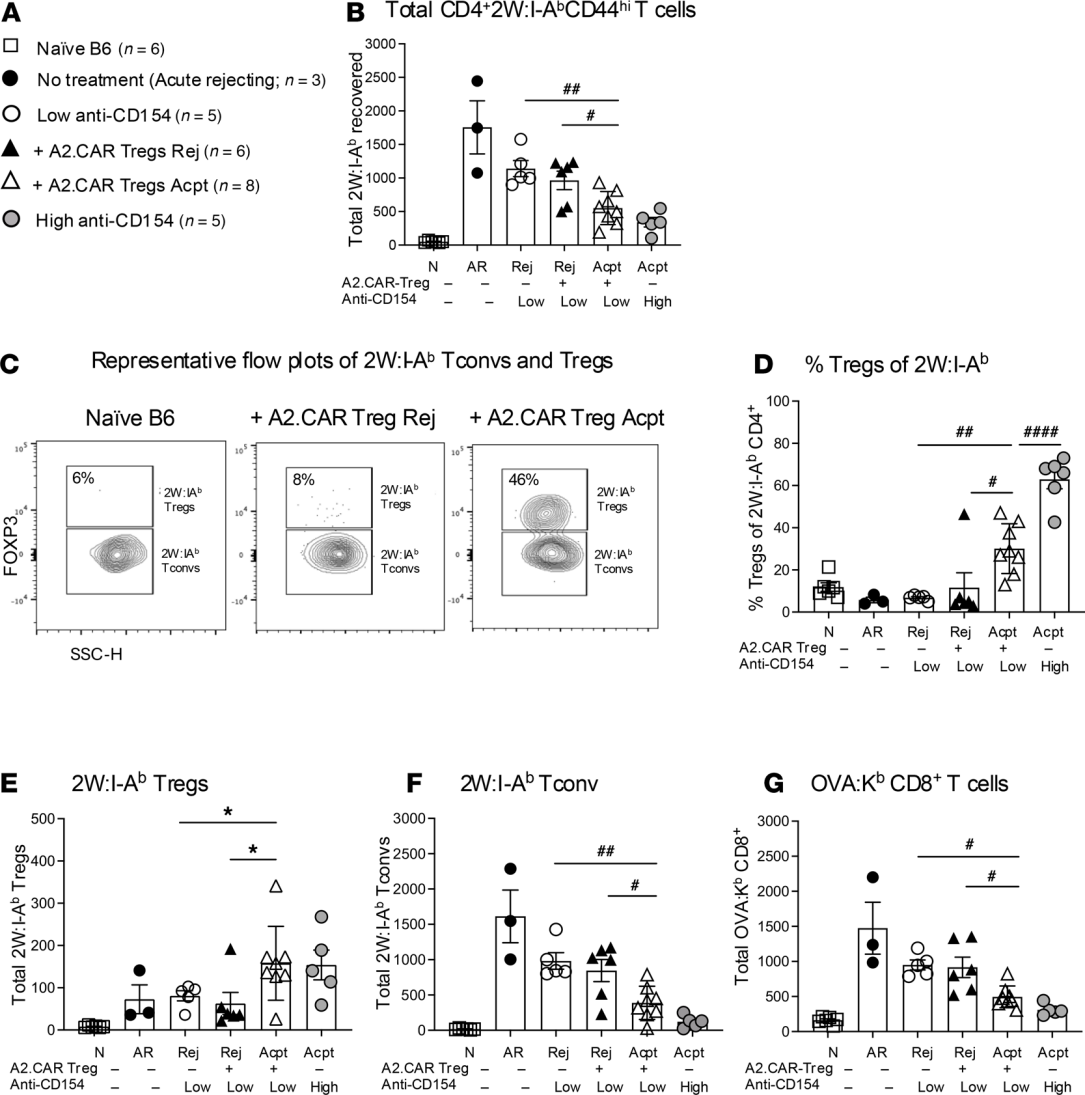
Graft acceptance in A2.CAR Tregs recipients is associated with expanded endogenous donor-specific Tregs and reduced T cell responses. (**A**) Experimental groups for **B** and **D**–**G**. (**B**) Number of CD4^+^2W:I-A^b^CD44^hi^ T cells recovered from SLOs. (**C**) Representative flow plots of FOXP3^pos^ of CD4^+^2W:I-A^b^CD44^hi^ T cells from SLOs of the indicated experimental groups and harvested on day 45–63 after HTx. (**D** and **E**) Frequency and total number of 2W:I-A^b^CD44^hi^ Tregs and of (**F**) 2W:I-A^b^CD44^hi^ T Tconvs and (**G**) OVA:K^b^CD8^+^ T cells. Each symbol represents 1 mouse. (A total of 7 Acpt recipients received 1.4 × 10^6^, and 1 Acpt at day 100 after HTx received 1 × 10^6^ A2.CAR Tregs.) Total 2W:I-A^b^CD4^+^ T cells and OVA:K^b^CD8^+^ T cells from SLOs normalized to 3 × 10^6^ events. Data are presented as mean ± SEM, and statistical significance was determined by 1-way ANOVA (#) and Mann-Whitney test (*). *P or ^#^*P* < 0.05; ^##^*P* < 0.01; ^####^*P* < 0.0001.

Donor grafts expressed A2, BALB/c alloantigens, and the model antigen 2W-OVA, which allowed us to track endogenous polyclonal 2W- and OVA-specific CD4^+^ and CD8^+^ T cells by utilizing 2W:I-A^b^ and OVA:K^b^ tetramers, respectively (Supplemental Figure 3). The experimental groups were Acpt or Rej HTx recipients receiving A2.CAR Tregs and low anti-CD154, and controls were naive B6, B6 HTx recipients with no treatment (acute rejecting, AR), or B6 HTx recipients receiving low- or high-dose anti-CD154 monotherapy (Figure 4A). Overall, the total number of CD4^+^2W:I-A^b^CD44^hi^ T cells recovered from the SLOs of Acpt recipients was significantly lower compared with Rej recipients (Figure 4B). Remarkably, the number of FOXP3^pos^2W:I-A^b^ Tregs recovered was significantly higher while the number of FOXP3^neg^2W:I-A^b^ Tconvs was lower in Acpt versus Rej recipients (Figure 4, C–F). Consequently, the percentage of FOXP3^pos^ of 2W:I-A^b^ cells was significantly higher in Acpt versus Rej recipients (35%–56% versus 5%–15%). The total number of OVA:K^b^CD8^+^ T cells was also significantly lower in Acpt recipients compared with Rej recipients (Figure 4G). These data demonstrate the ability of A2.CAR Tregs to mediate infectious tolerance by reshaping host immunity toward allograft tolerance. Since the A2.CAR Tregs express endogenous TCR, we quantified 2W:I-A^b^Thy1.1^+^ cells and observed that their frequency was extremely low and there was no difference between Rej and Acpt groups (Supplemental Figure 4).

Flow cytometric analysis of 2W:I-A^b^ Tregs and Tconvs, as well as OVA:K^b^CD8^+^ T cells from Acpt versus Rej recipients of A2.CAR Tregs + anti-CD154 revealed that they were phenotypically comparable (Supplemental Figures 5–7). These markers, namely FR4, CD73, PD-1, and SLAMF6, were able to distinguish these T cells from control rejection (untreated or low anti-CD154) versus control acceptance (high anti-CD154). Thus, while endogenous donor-specific CD4^+^ and CD8^+^ T cells expanded in Rej recipients, their phenotype was unexpectedly conserved in Rej and Acpt recipients.

**Figure 5:**
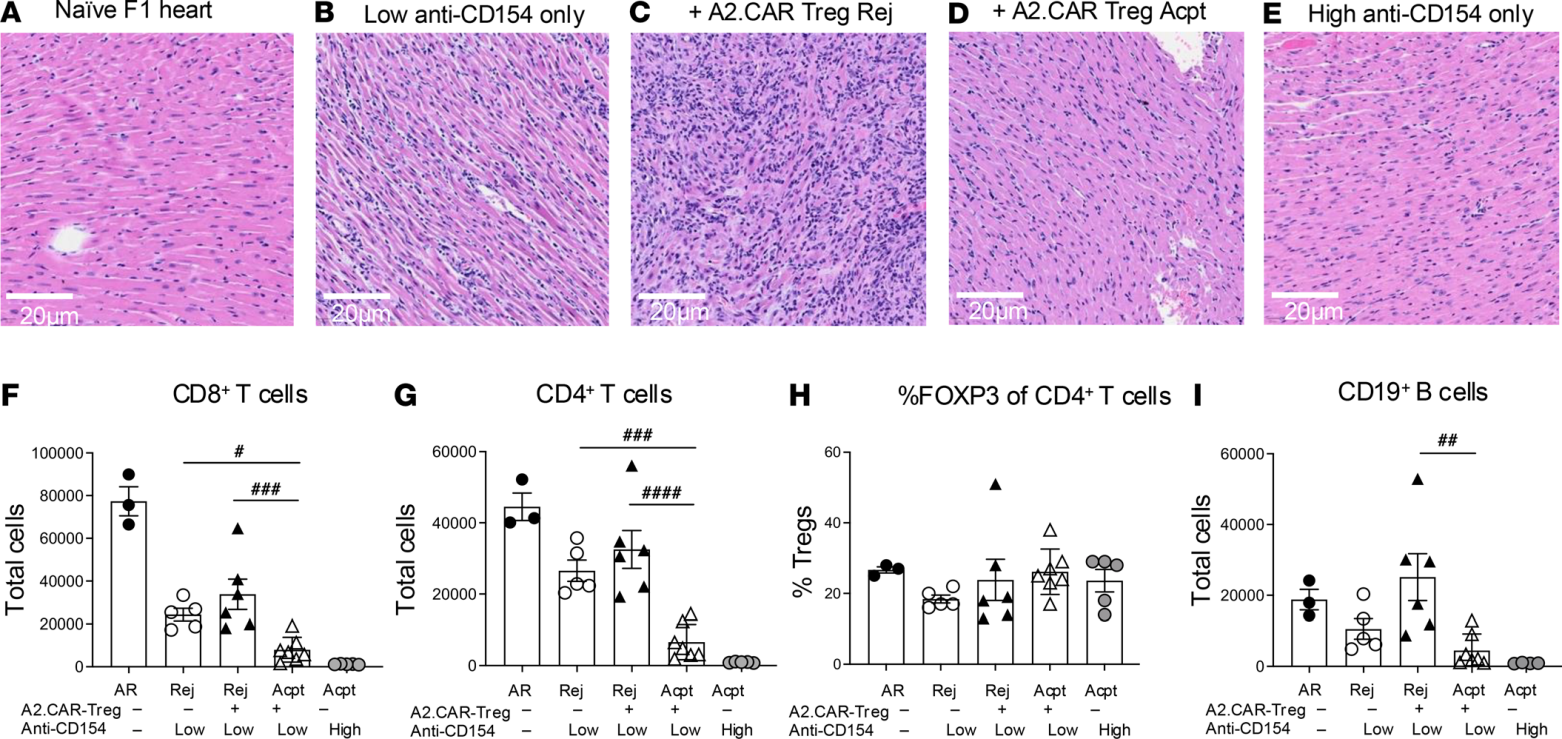
Graft acceptance in A2.CAR Treg recipients is associated with reduced accumulation of endogenous T and B cells in heart allografts. (**A**–**E**) Representative H&E-stained histological sections (scale bar: 20 μm) of naive or transplanted heart allografts from indicated groups sacrificed on days 45–63 after HTx. (**F**–**I**) Cells infiltrating the allograft were analyzed by flow cytometry. Total graft-infiltrating (**F**) CD8^+^ T cells, (**G**) CD4^+^ T cells, (**H**) percentage Tregs of CD4^+^ T cells, and (**I**) CD19^+^ B cells were normalized to 1 × 10^6^ events. Each symbol represents 1 mouse. Data are presented as mean ± SEM, and statistical significance was assessed by 1-way ANOVA. ^#^*P* < 0.05; ^##^*P* < 0.01; ^###^*P* < 0.001; ^####^*P* < 0.0001.

Histology of transplanted hearts (Figure 5, A–E) verified that lymphocyte infiltration was minimal in Acpt but abundant in Rej grafts. Flow cytometry performed to quantify the endogenous (Thy1.1^–^) T cells and B cells accumulating in allografts verified that endogenous T cell and B cell infiltration was significantly increased in Rej versus Acpt grafts (Figure 5, F–I). Taken together, these data illustrate the ability of A2.CAR Tregs to synergize with anti-CD154 to mediate infectious tolerance, leading to controlled endogenous donor-specific CD4^+^ and CD8^+^ T cell responses, both systemically and in the graft, and to improved allograft survival. In contrast, no infectious tolerance was observed in A2.CAR Treg recipients who rejected their grafts.

### A2.CAR Tregs inhibit anti-A2 and anti-donor MHC B cell responses.

**Figure 6:**
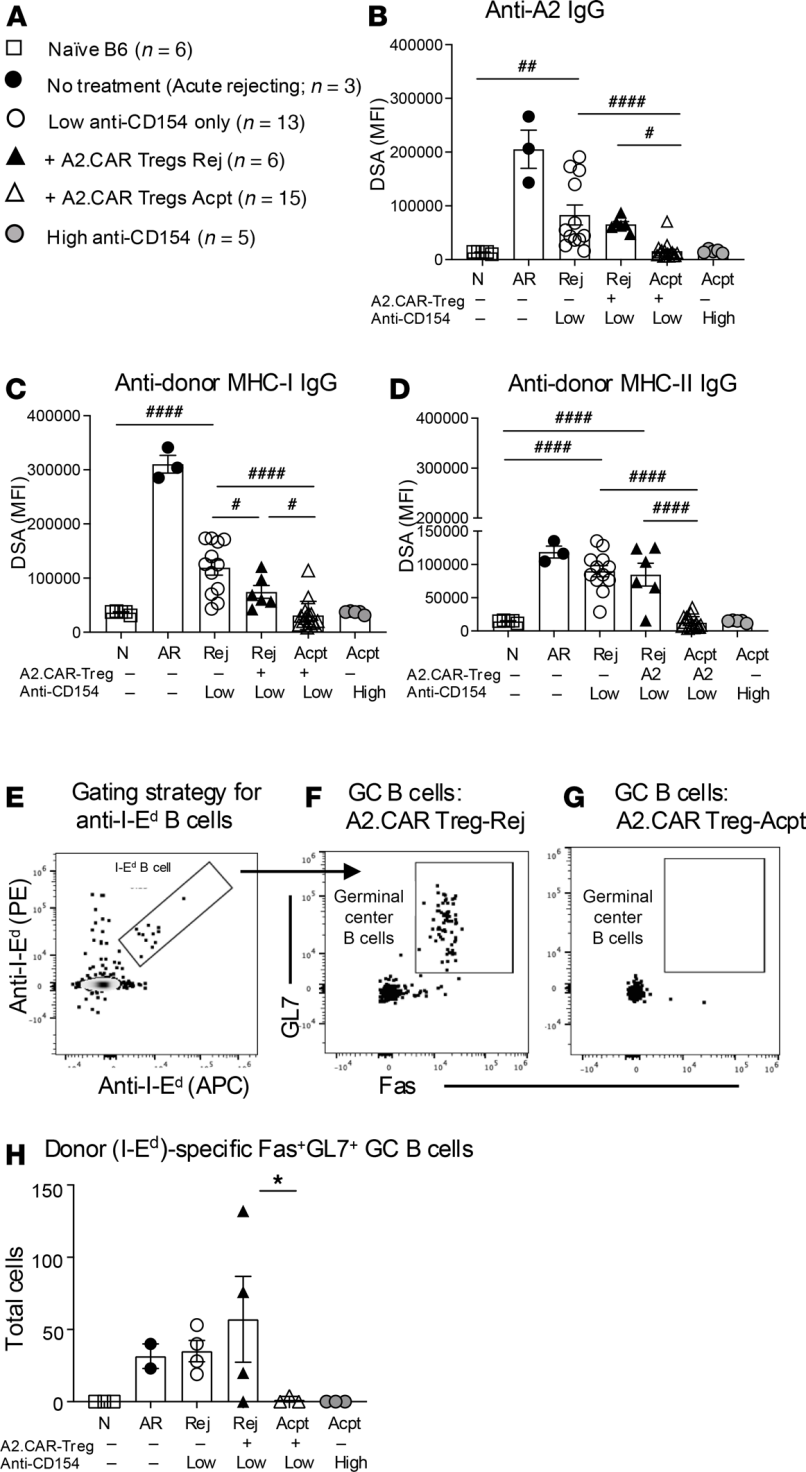
A2.CAR Tregs synergize with low anti-CD154 to suppress non-A2 donor-specific B cell responses. (**A**) Symbols for **B**–**D** and **H**. (**B**) Quantification of IgG specific for A2, (**C**) donor MHC-I IgG, and (**D**) donor MHC-II at day 45 after HTx. (**E**–**G**) Gating strategy to track donor MHC-II I-E^d^–specific GC B cells (Fas^+^GL7^+^) from (**F**) +A2.CAR Treg Rej or (**G**) +A2.CAR Treg Acpt. (**H**) Total GC B cells (Fas^+^GL7^+^ of I-E^d^) recovered from draining lymph nodes (normalized to 2 × 10^6^ events). Each symbol represents 1 mouse. Data are presented as mean ± SEM, and statistical significance was determined by 1-way ANOVA (#) and Mann-Whitney test (*). *P or ^#^*P* < 0.05; ^##^*P* < 0.01; ^####^*P* < 0.0001. DSA, donor-specific antibody.

Prior studies reported that A2.CAR Tregs suppressed anti-A2 IgG responses in single-antigen-mismatched transplant models (21), but the ability to control non-A2 IgG was unknown. We found that in Acpt recipients, A2.CAR Tregs significantly suppressed DSA responses to HLA-A2 as well as to BALB/c MHC class I (MHC-I) (K^d^, L^d^) and MHC-II (I-E^d^, I-A^d^) (Figure 6, A–D). Indeed, IgG levels to all 3 specificities were comparable to recipients treated with high anti-CD154 or naive mice. In contrast, Rej recipients had higher levels of DSA by day 45 post-HTx, though anti-donor MHC-I titers remained significantly lower compared with recipients of low anti-CD154 without A2.CAR Tregs.

Furthermore, by utilizing donor MHC-II I-E^d^ tetramers, we tracked endogenous I-E^d^–specific B cells and characterized their phenotype (Figure 6, E–G). Recipients with Acpt allografts had decreased germinal center B (GC: Fas^+^GL7^+^) responses compared with recipients with Rej allografts or recipients treated with low anti-CD154 only (Figure 6H). Thus, these data show that A2.CAR Tregs were able to synergize with anti-CD154 to control endogenous DSA responses that are specific not only for A2 but also for donor MHC-I and -II.

## Discussion

CAR Tregs are already being tested clinically, despite limited experimental evidence for how to effectively combine them with immunosuppression to promote the stable acceptance of multiple-antigen-mismatched allografts in immunocompetent recipients (22). Our study provides major advances in both of these areas by providing evidence that in combination with CD154 blockade, A2.CAR Tregs extend the survival of haplo-mismatched heart allografts. Importantly, treatment with CAR Tregs specific for a single donor (A2) antigen suppressed non-A2 donor-reactive CD4^+^ T cell, CD8^+^ T cell, and B cell responses and promoted the expansion of FOXP3^+^ cells within the endogenous donor-specific (2W:I-A^b^) CD4^+^ T cell population. These data build upon evidence that A2.CAR Treg therapy promotes infectious tolerance in a model of islet transplantation (27) and support the conclusion that infusion of a single dose of donor-specific CAR Tregs can broadly reshape allograft immunity to promote tolerance. Since the general consensus is that long-term allograft tolerance is mediated by endogenous Tregs with indirect specificity (28, 29), evidence that infused A2.CAR Tregs can promote the expansion of endogenous Tregs with indirect specificity is a key advance. A caveat is that the A2.CAR Tregs also express endogenous TCR. Although the frequency of 2W:I-A^b^ tetramer–binding CD4^+^Thy1.1^+^ cells was extremely low, we cannot exclude the possibility of endogenous TCRs expressed by the A2.CAR Tregs contributing to the regulating of anti-donor immune responses.

It was previously reported that A2.CAR Tregs can suppress A2-specific B cells and antibody production (21), but their ability to control antibody responses to other alloantigens was unknown. Here, we showed that both A2-specific and IgG responses specific for donor MHC-I and -II were successfully controlled and that the differentiation of donor MHC-specific B cells into GC B cells was inhibited compared with recipients treated with low anti-CD154 alone. Whether this control of donor-specific humoral immunity was mediated by A2.CAR Tregs preventing the generation of T follicular helper cells, promoting the differentiation of regulatory T follicular cells, or both will require further investigation (30). Nevertheless, the observation that infectious tolerance extends to B cell responses is important, as DSA has been implicated in chronic allograft rejection in the clinic (31–33).

It is notable that the beneficial effects of anti-CD154 and A2.CAR Treg therapy occurred in only approximately half of the recipients, even with the same batch of CAR Tregs and with recipients being cohoused before and after transplantation. This finding aligns with other studies, where the efficacy of adoptively transferred Tregs in models of islet, heart, and skin transplants was also heterogenous, and graft survival rates did not exceed 80% at day 100 posttransplantation (29, 34, 35). Our investigations into mice where allograft survival was not prolonged revealed that, as early as 2 weeks after adoptive transfer, there was a significant expansion of A2.CAR Tregs in the blood, and SLOs, of Rej compared with Acpt recipients (Figure 2), though the percentage of cells that were FOXP3^pos^ remained comparable. We also observed higher ratios of FOXP3^neg^/FOXP3^pos^ A2.CAR T cells within Rej versus Acpt grafts at the time of sacrifice (45–63 days post-HTx), thus emphasizing the need to investigate events within the allograft. This accumulation of FOXP3^neg^ A2.CAR T cells could be due to preferential expansion of FOXP3^neg^ cells present in the infused product, which contained about 3%–5% FOXP3^neg^ cells, or destabilization of FOXP3^pos^ into FOXP3^neg^ cells (36–39). Inflammatory cytokines, such as IL-6, produced early posttransplantation may have contributed to either of these processes, as previously reported for polyclonal Tregs (40, 41). Future investigations into the origin of FOXP3^neg^ A2.CAR Tregs, and why A2.CAR Tregs failed to reshape host immunity in some mice, will be important.

Tregs control APCs and alloreactive T and B cells through a variety of mechanisms, including the expression of coinhibitory molecules, trogocytosis of stimulatory molecules on APCs, secretion of immunoregulatory cytokines, depletion of IL-2, and expression of ectonucleotidases, CD73 and CD39, which catabolize ATP into immunosuppressive adenosine (42–46). Furthermore, FR4, a surface receptor for folic acid (vitamin B_9_), has been implicated in mediating Treg function and is highly expressed on antigen-responsive Tregs (47). We observed that HTx acceptance was associated with FOXP3^pos^ A2.CAR Tregs with upregulated FR4 and CD73, raising the possibility that these represent CAR Tregs with regulatory function.

In summary, our study addresses several mechanistic gaps in knowledge. However, we acknowledge that more work is required to fully understand the state of graft acceptance in mice treated with CAR Tregs plus anti-CD154 therapy. While A2.CAR Tregs synergized with anti-CD154 to promote the development or expansion of endogenous, antigen-specific Tregs, consistent with the concept of infectious tolerance (5, 28, 48), many questions remain. First, whether this is driven by A2.CAR Tregs secreting antiinflammatory cytokines, such as IL-10, IL-35, and TGF-β, which act on T cells, or by acting on APCs has yet to be determined. Second, we observed increased ratios of FOXP3^neg^/FOXP3^pos^ A2.CAR Tregs in Rej grafts, but very few A2.CAR Tregs infiltrated Acpt grafts. The inflammatory milieu unique to the Rej graft driving the recruitment of A2.CAR Tregs and the increase in FOXP3^neg^/FOXP3^pos^ ratios remains to be elucidated. Third, it is unclear if B cell suppression is due to reduced T follicular helper cell help or to direct A2.CAR Treg binding to cross-dressed HLA-A2 on B cells (49) and warrants additional study. Overall, our study supports the clinical testing of CAR Treg therapy in combination with CD154 pathway blockade. Heterogeneity in efficacy underscores the need for additional mechanistic studies to guide iterative improvement of the CAR Treg product to maximize its potency and consistency.

## Methods

### Sex as a biological variable.

Our study was conducted only in female recipients, as it difficult to perform surgeries on and cohouse male recipients. Donor hearts and the CAR Tregs were also from female mice. We acknowledge this is a limitation, although we anticipate the results of this study to apply to male recipients.

### Mice.

HTx recipients were 8- to 12-week-old female B6 (H-2^b^) mice purchased from Harlan Sprague Dawley. Heart donor (A2.2W-OVA.F1) mice were generated by crossing 2W-OVA.BALB/c males (50) (2W-OVA.B6 mice were a gift from James J. Moon, Massachusetts General Hospital, Harvard Medical School, Boston, Massachusetts, USA) with B6.HLA-A*0201 females (Taconic Biosciences) to generate 2W-OVA.BALB/c mice. B6-FOXP3^gfp^ Thy1.1 mice, used for generating Tregs, were bred in-house (18, 21).

### Generation of A2.CAR Tregs.

A2.CAR Tregs were generated as described previously (9, 18). Briefly, lymph nodes and spleen from female B6-FOXP3^gfp^ Thy1.1 mice were collected, and CD4^+^ T cells were isolated by negative selection (STEMCELL Technologies). Tregs were sorted as live CD4^+^CD8^–^Thy1.1^+^FOXP3^gfp+^ or CD4^+^CD8^–^Thy1.1^+^CD25^+^CD62L^+^ cells using a Moflo Astrios cell sorter (Beckman Coulter), stimulated with anti-CD3/CD28 Dynabeads (Thermo Fisher Scientific), and expanded with recombinant human IL-2 (1,000 U/mL; Proleukin) in the presence of rapamycin (50 nmol/L; MilliporeSigma). After 2 days, cells were transduced with a retrovirus encoding an A2-specific, CD28-containing, second-generation A2.CAR (Supplemental Figure 1A). Dynabeads were removed on day 7, and CAR expression and Treg purity were determined (Supplemental Figure 1, B and C). Cells were cryopreserved prior to injection as a cell therapy.

### Adoptive transfer of A2.CAR Tregs and heterotopic HTx.

A2.CAR Tregs were administered on the day prior to HTx at the indicated doses via the intravenous (i.v.) route. Heterotopic HTx was performed as described (51), by anastomosing female donor A2.2W-OVA F1 hearts to the inferior vena cava and aorta in the peritoneal cavity of female B6 recipients. Anti-CD154 (MR1, BioXCell) was injected i.v. on the day of HTx at the indicated doses. Allograft survival was monitored twice weekly starting at POD 7 by transabdominal graft palpation and scored on a scale of 0 to 4. Allograft rejection was defined as the last day of palpable heartbeat.

### Harvesting of allograft and SLOs and tissue processing for flow cytometry.

Mice were euthanized at the indicated times post-HTx, and heart grafts were harvested and perfused with cold PBS, disintegrated, and digested by incubating for 20 minutes at 37°C with 2 mg/mL collagenase type IV (MilliporeSigma). SLOs comprising spleens and pooled lymph nodes (brachial, inguinal, axillary, and mediastinal) were harvested, and single-cell suspensions were obtained from passing through a 40 μm cell strainer (Corning Inc.). Red blood cells were lysed via 2- to 3-minute incubation with ammonium chloride-potassium lysis buffer (Quality Biological). T cells were enriched with a pan–T cell isolation kit II (Miltenyi Biotec) and passed through LS columns on a QuadroMACS separator (Miltenyi Biotec), followed by elution with MACS buffer (2% FBS + 2 mM EDTA).

### Tetramer, fluorescent antibodies, and reagents.

After T cell enrichment, cells were stained with Fixable Aqua Live/Dead staining (Thermo Fisher Scientific), then Fc blocked (anti-CD16/32, S17011E, BioLegend). Tetramer staining was carried out for 40–45 minutes at 4°C; APC- and PE-conjugated (NIH Tetramer Facility) 2W (EAWGALANWAVDSA):I-A^b^ tetramers were used to track alloreactive CD4^+^ and OVA (SIINFEKL):H-2K^b^ tetramers for CD8^+^ T cells. pCons-CDR1 (FIEWNKLRFRQGLEW):I-E^d^ tetramers (PE-conjugated) and H2-K^b^ (SIINFEKL) decoy (AF647-PE-conjugated) tetramers were used to identify donor MHC-II–reactive B cells as previously described (52, 53).

Antibodies used to define the phenotype of T cells included Invitrogen: CD44-BUV737 (IM7), CD8α-PE (53-6.7); Abcam: c-Myc–AF488 (Y69); BioLegend: IgG2a- PECy7 (RMG2a-62), IgD-BV605 (11-26c.2a), T and B cell activation marker–FITC (GL7), CD73-BV605 (TY/11.8), PD-1–APC-Cy7 (RMP1-30), TIGIT-PECy7 (1G9), LAG3-BV785 (C9B7W), CD62L APC (MEL-14); BD Biosciences: CD19-BV421 (1D3), CD95- BUV737 (Jo2), CD90.2-BUV395 (53-2.1), CD4-BV510/BV786 (GK1.5), CD4-BUV496 (RMA.5), Thy1.1- BUV496 (HIS51), CD8-BUV805 (53-6.7), FR4-BV421 (12A5), FOXP3–Alexa Fluor 532/PECy7 (FJK-16s), CD25 BV421 (PC61); and Ablab UBC: c-Myc–AF647/AF488 (9E10). Antibodies used to exclude non-T or non-B cells were CD11c-BUV661 (N418), F4/80-BUV661 (T45-2342), NK1.1-2 BUV661 (PK136), and TER119-BUV661 (TER-119) (BioLegend).

### Multiplex bead assay to measure DSA.

Multiplex streptavidin beads (PAK-5067-10K; Spherotech) were coated with biotinylated MHC-I (K^d^, L^d^), MHC-II (I-E^d^, I-A^d^), and HLA-A*0201 monomers (NIH Tetramer Facility). Sera were incubated with pooled MHC-I–, MHC-II–, and HLA-A2–coated beads at 4°C for 1 hour. Beads were washed and then stained with FITC-conjugated anti-mouse IgG (1070-09; Southern Biotech). The MFI of DSA binding to the multiplex beads was determined by flow cytometry (Novocyte Quanteon, Agilent) (52).

### Statistics.

Statistical significance analyses were performed using GraphPad Prism. Graft survival significance was assessed using Kaplan-Meier/Mantel-Cox log-rank tests. Statistical differences between experimental groups were determined by 1-way ANOVA or Mann-Whitney unpaired *t* test. *P* values ≤ 0.05 were considered statistically significant.

### Study approval.

All animal experiments were approved by the Institutional Animal Care and Use Committee at the University of Chicago or University of British Columbia and adhered to the standards of the NIH *Guide for the Care and Use of Laboratory Animals* (National Academies Press, 2011) or Canadian Council of Animal Care.

### Data availability.

Values of all data shown can be found in the Supporting Data Values file.

## Author contributions

SSD designed the study, conducted the experiments, analyzed data, and wrote the manuscript. IRS, MS, and MM generated A2.CAR Tregs. DY performed the HTx. IS and GEH provided help with data analysis. MLA provided critical feedback on the study design. MKL and ASC designed the study and wrote the manuscript. All authors edited and provided feedback on the manuscript.

## Supplementary Material

Supplemental data

Supporting data values

## Version 1 04/08/2025

Electronic publication
