# Supplementary material for: CAR Treg synergy with anti-CD154 promotes infectious tolerance and dictates allogeneic heart transplant acceptance
Source: JCI Insight. 2025 Apr 8;10(7):e188624. doi: 10.1172/jci.insight.188624 (PMC11981628; doi:10.1172/jci.insight.188624)
Supplement: Supplemental data [file jciinsight-10-188624-s054.pdf]

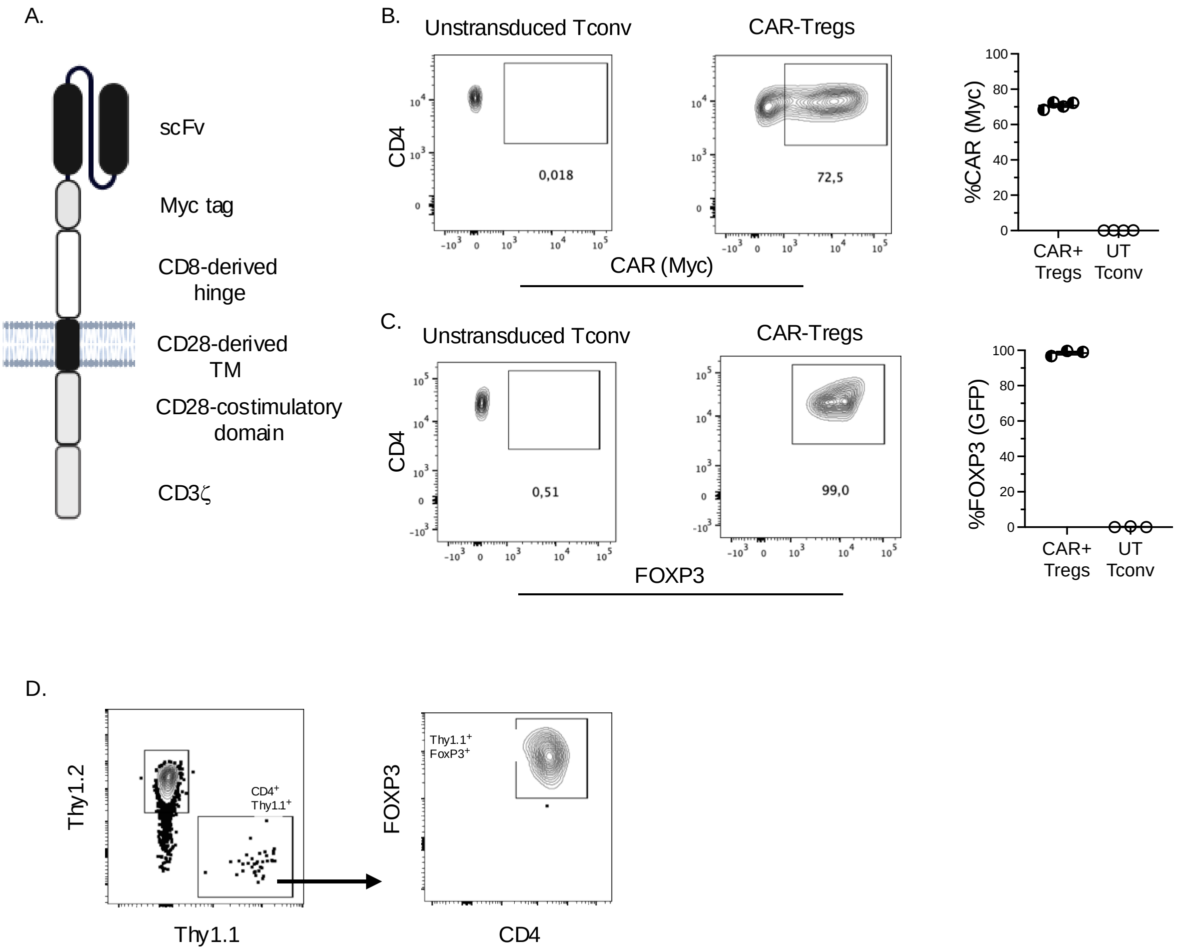

Supplementary figure 1: **CAR structure and generation of A2.CAR Tregs. (A)** Schematic diagram indicating domains of the second generation HLA-A2-specific CAR used in this study. Thy1.1^{+}FOXP3^{+} Tregs were sorted, activated, transduced and expanded as described in Methods. Parallel cultures of untransduced (UT) Tconv were used for gating controls. After 7 days, the proportions cells expressing **(B)** Myc and **(C)** FOXP3^{gfp} were determined. **(D)** Representative flow plots showing the gating strategy to track Thy1.1+ A2.CAR Tregs in the blood.

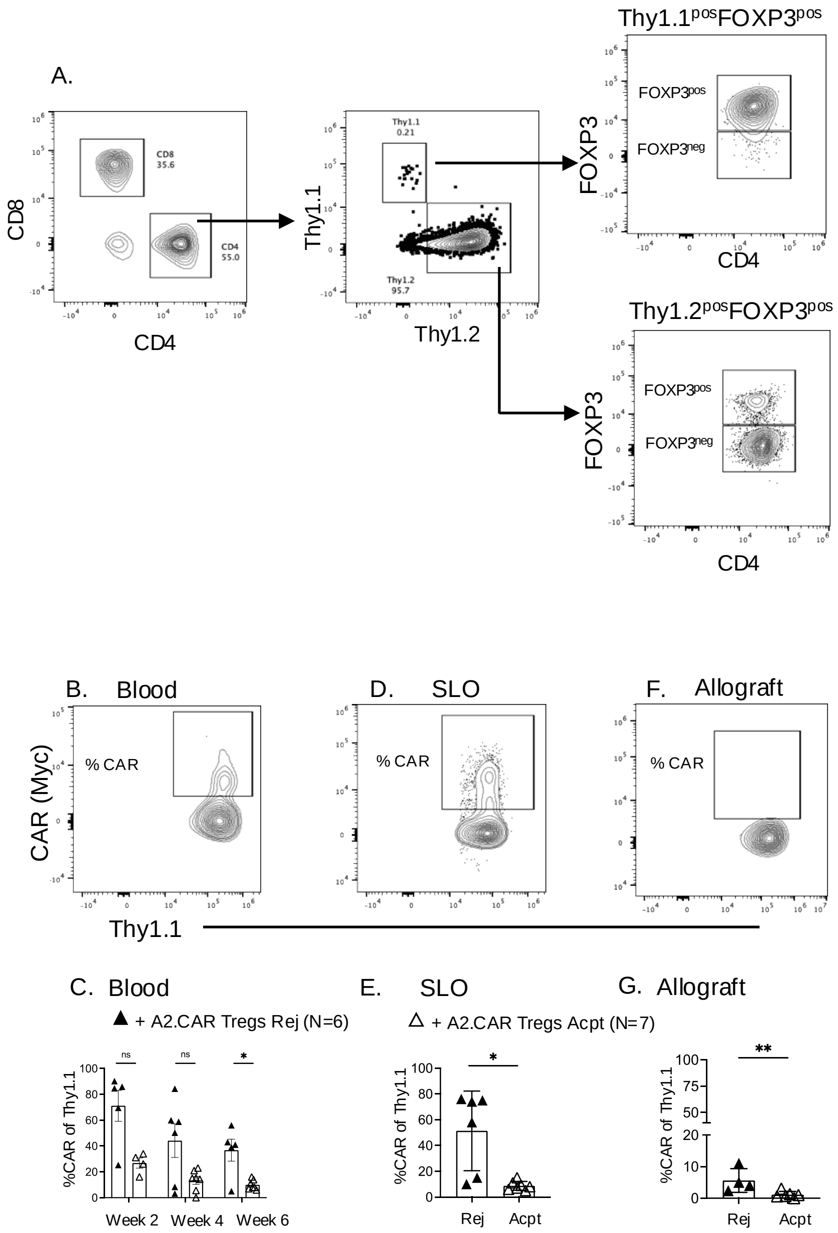

Supplementary Figure 2: (A) Gating strategy to track Thy1.1^{+}FOXP3^{+} versus endogenous Thy1.2^{+}FOXP3^{+} cells from the SLO. **(B-G)** Expression of CAR (Myc) by Thy1.1^{+} A2.CAR Tregs from the circulation, SLO and allografts at rejection (Rej; filled triangle) or acceptance (Acpt; open triangle). **(B,D,F)** Representative flow plots and (**C,E,G**) frequency of CAR (Myc) expression by Thy1.1^{+} cells in the blood, SLO and allograft for HTx recipients treated with A2.CAR Tregs + low dose anti-CD154. Each dot represents one mouse, data are presented as mean ± SEM, and statistical differences were assessed by Mann-Whitney test. *$P<0.05$; **$P<0.01$

A.

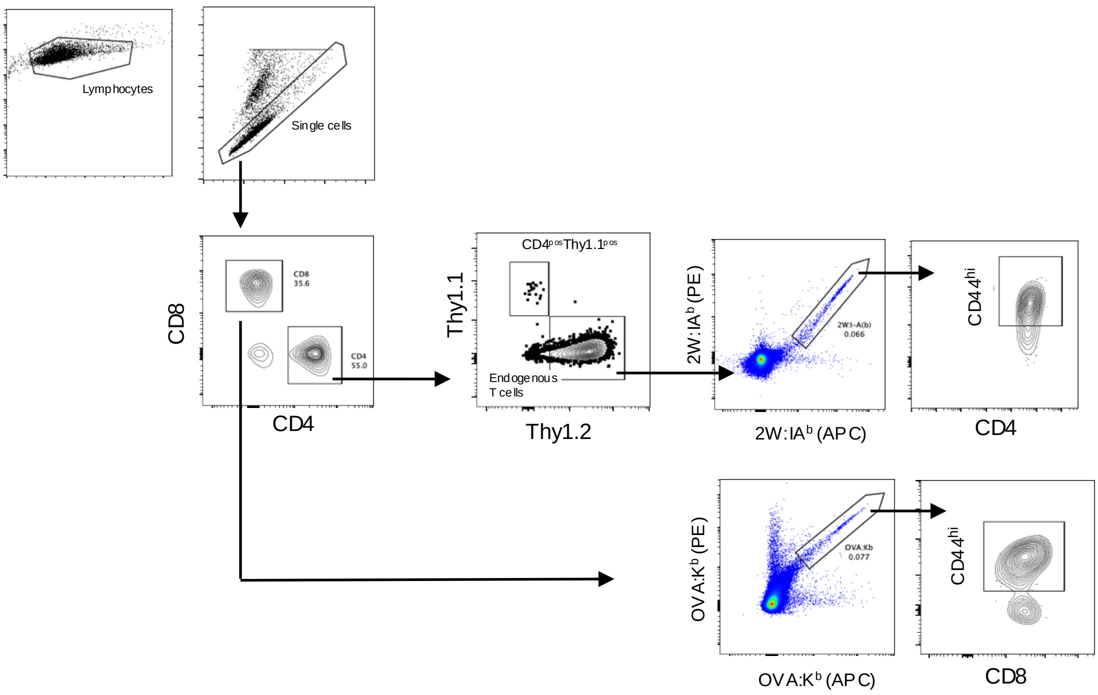

Supplementary Figure 3: Gating strategy to track 2W:I-A^b $CD4^+CD44^{hi}$ and OVA:K^b $CD8^+CD44^{hi}$ T cells using a single tetramer double fluorochrome approach.

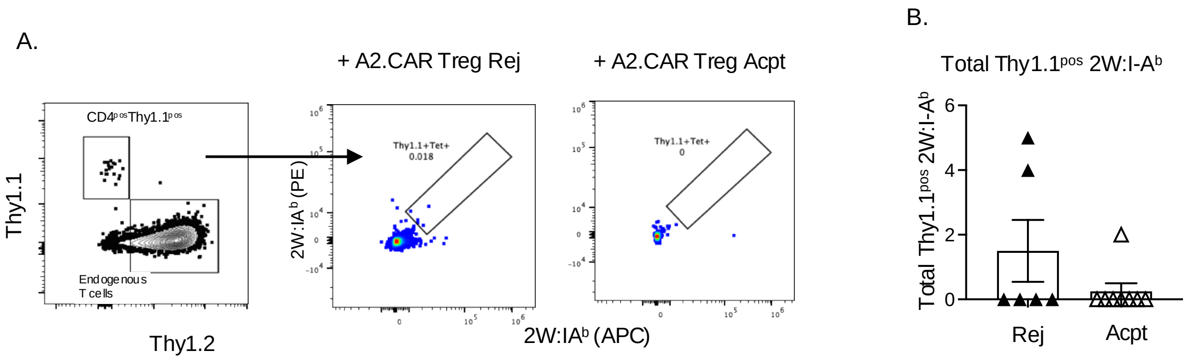

Supplementary Figure 4: A) Gating strategy to track 2W:I-A^b tetramer binding $CD4^+Thy1.1^+$ cells. **B)** Total 2W:I-A^b $CD4^+Thy1.1^+$ T cells from the SLO of Rej and Acpt mice.

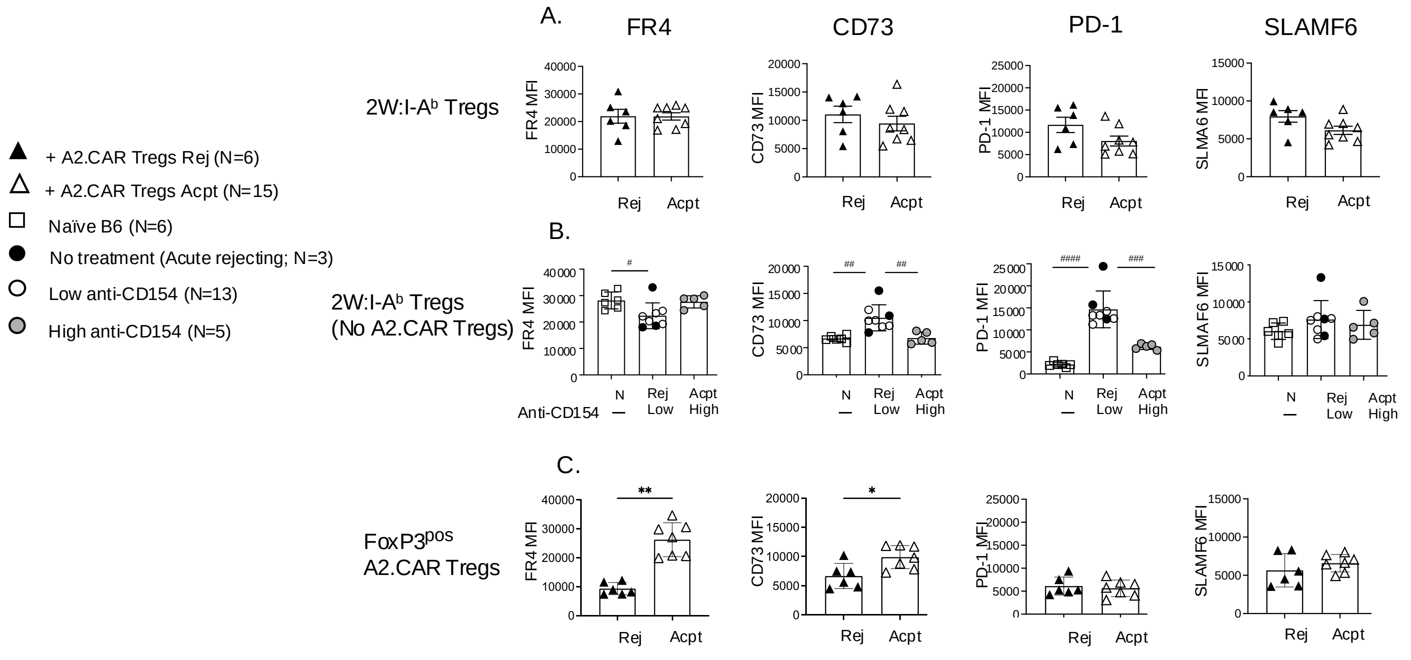

Supplementary Figure 5: Phenotype of endogenous 2W:I-A^b Tregs and FoxP3$^+$A2.CAR Tregs at rejection (Rej) and acceptance (Acpt): Phenotype of **(A)** 2W:I-A^b Tregs in Rej vs Acpt recipients of A2.CAR Tregs + low dose anti-CD154 and **(B)** 2W:I-A^b Tregs from HTx recipients that received low or high dose anti-CD154 but no A2.CAR Tregs. Phenotype of **(C)** FoxP3^{pos} A2.CAR Tregs in Rej vs Acpt HTX recipients of A2.CAR Tregs + low dose anti-CD154, replicated from Fig 1C for ease of comparison. Each symbol represents one mouse. Data are presented as mean ± SEM and statistical significance determined by 1 way ANOVA (#) and Mann Whitney test (*). *P or $^{\#}$P<0.05; **P or $^{\#\#}$P <0.01; $^{\#\#\#}$P <0.001; $^{\#\#\#\#}$P<0.0001.

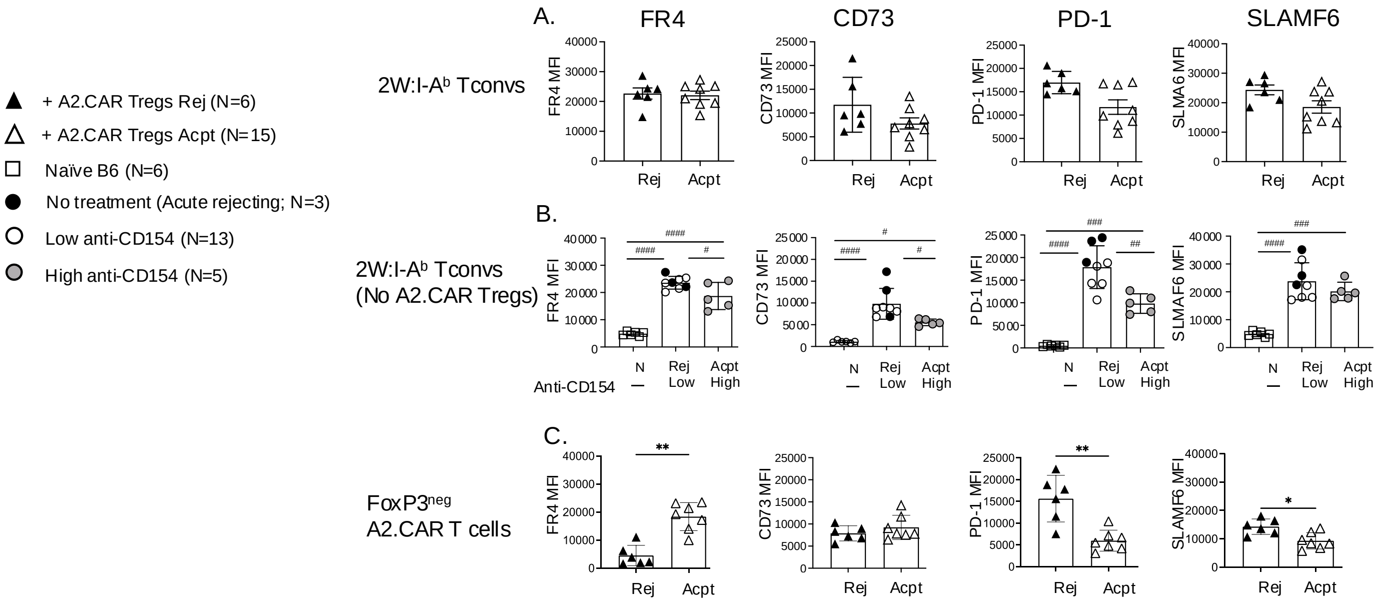

Supplementary Figure 6: Phenotype of FOXP3^{neg}A2.CAR T cells and endogenous 2W:I-A^{b} Tconvs at rejection (Rej) and acceptance (Acpt): **(A)** Phenotype of FOXP3^{neg} 2W:I-A^{b} Tconvs in Rej vs Acpt HTX recipients of A2.CAR Tregs + low dose anti-CD154, **(B)** FOXP3^{neg} 2W:I-A^{b} Tconvs in HTx recipients treated with low dose anti-CD154 without A2.CAR Tregs and **(C)** FOXP3^{neg} A2.CAR T cells in Rej vs Acpt HTX recipients of A2.CAR Tregs + low dose anti-CD154, replicated from Fig 1D for ease of comparison. Each symbol represents one mouse. Data are presented as mean ± SEM and statistical significance determined by 1 way ANOVA (#) and Mann Whitney test (*). *P or $^{\#}$P<0.05; **P or $^{\#\#}$P <0.01; $^{\#\#\#}$P <0.001; $^{\#\#\#\#}$P<0.0001.

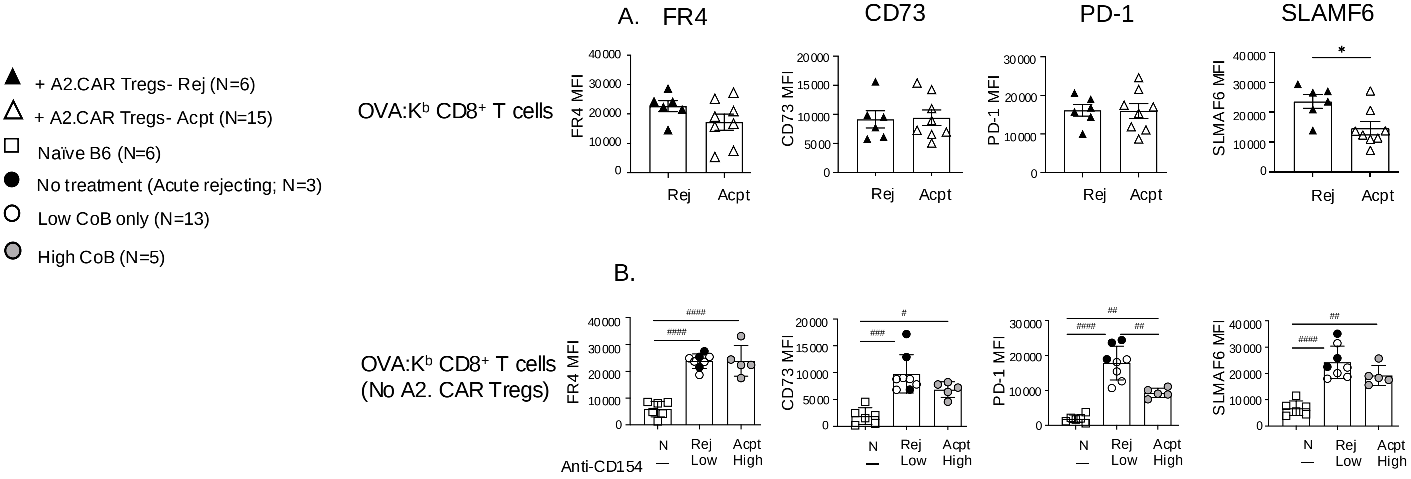

Supplementary Figure 7: Phenotype of endogenous OVA:K^b CD8 T cells at rejection (Rej) vs acceptance (Acpt). **(B-E)** Phenotype of OVA:K^b CD8$^+$ T cells in Rej vs Acpt HTX recipients of A2.CAR Tregs + low dose anti-CD154. **(F-I)** Phenotype of 2W:I-A^b Tregs in HTx recipients treated with low dose anti-CD154 without A2.CAR Tregs. Each symbol represents one mouse. Data are presented as mean ± SEM and statistical significance determined by 1-way ANOVA (#) and Mann Whitney yest (*). *P or $^{\#}$P<0.05; **P or $^{\#\#}$P <0.01; $^{\#\#\#}$P <0.001; $^{\#\#\#\#}$P<0.0001.
